# Linking health literacy to medication adherence in chronic disease management from a community pharmacy perspective

**DOI:** 10.3389/fpubh.2026.1865416

**Published:** 2026-07-17

**Authors:** Lobna Gharaibeh, Mohammad Alhariri, Saja Smadi, Tala Alalem, Huda Al Riyati, Rahaf Abu-safi, Rama Abu-Al-Roos, Wisam Hilow, Roaa Hamideh, Rana Abu-Farha

**Affiliations:** 1 College of Pharmacy, Amman Arab University, Amman, Jordan; 2 Department of Clinical Pharmacy, Jordan University of Science and Technology, Irbid, Jordan; 3 College of Pharmacy, Aqaba University of Technology, Aqaba, Jordan; 4 Department of Clinical Pharmacy and Therapeutics, Faculty of Pharmacy, Applied Science Private University, Amman, Jordan

**Keywords:** adherence, community pharmacist, health literacy, HLS-EU-Q16, Jordan, Morisky medication adherence scale (MMAS-8)

## Abstract

**Introduction:**

Health literacy is an important health determinant that is associated with adherence to medication. This complex relationship affects the control of chronic medical conditions and may offer opportunities that impact the role of community pharmacist.

**Methods:**

This is a cross-sectional study conducted through interviews with patients in the community pharmacy settings. The adherence to medication was assessed using the validated Arabic version of the Morisky Medication Adherence Scale (MMAS-8). Health literacy was evaluated using the validated Arabic version of the Health Literacy Survey European Questionnaire’s short version (HLS-EU-Q16).

**Results:**

Half of the participants exhibited low adherence (*n* = 272, 54.2%), 27.3% demonstrated medium adherence (*n* = 137), and one-fifth achieved high adherence (*n* = 93, 18.5%). Only one fifth of the participants received counselling at every refill and few participants reported experiencing private counselling in community pharmacies. Most participants demonstrated sufficient health literacy (*n* = 330, 65.7%). A smaller proportion were classified as having problematic health literacy (*n* = 123, 24.5%), while inadequate health literacy was observed in 49 participants (9.8%). Patients with inadequate health literacy had the highest proportion of low adherence. Significant predictors of adherence included health literacy, geographical region, and individuals’ perception of their health.

**Conclusion:**

Inadequate health literacy was associated with low adherence. Health literacy, geographic/regional location, and health status were significant predictors of adherence. Community pharmacists play an important role in improving health literacy through interventions that can be implemented in an individualized manner taking into consideration the characteristics of the patients.

## 1 Introduction

Chronic medical conditions like diabetes, cardiovascular diseases and cancer are on the rise. They will continue to be responsible for a large percentage of morbidity and mortality. As the population lives longer, these diseases will increase in prevalence and cost (1). Poor control of many diseases is primarily caused by non-adherence and stopping the medication (2). Medication is the last step in the long costly healthcare process that involves physician visits, diagnostic tests and procedures, and finally drug dispensing. Lack of adherence deprives the patients from the benefits of medication and leads to waste of resources. In hypertensive patients, non-adherence is high and ranges from 55.5% in self-reporting and 46.6% in pill counting methods (3). Several factors exert an effect on adherence to medication, which renders the process complex and multifactorial. Good communications with the patient and providing appropriate information on their medical condition and drugs is pivotal in improving their adherence (4).

Health literacy is the ability of an individual to acquire, process, and understand information related to basic health and healthcare services (5). Health literacy is related to issues such as education, income, and accessible healthcare that have an impact on prevention and control of disease (6). The existing evidence on the relationship between health literacy and adherence is controversial and conflicting. Many studies demonstrated a positive correlation between heath literacy and adherence (7–9). Guo et al. (10) in a cross-sectional study revealed a positive relationship between health literacy/social support and adherence in patients with hypertension, they proposed that adherence can be improved through these concepts. In diabetic patients, Mohammi et al. (11) reported a positive correlation between functional/critical literacy and adherence but a negative one with communicative literacy. Jia et al. (12) evaluated the relationship between health literacy and adherence in a large sample of 4,166 older adults. Low health literacy scores were negatively associated with medication adherence. However, this association disappears in older adults with cognitive impairment.

Hyvert et al. (13) in a recent systematic review examined 27 studies, 9 reported no relationship, one study confirmed a negative relationship, 14 revealed a positive relationship, and 3 showed mixed results. They confirmed that the relationship between health literacy and medication adherence is still not clear and other factors exert an effect on adherence. Similarly, in type 2 diabetes mellitus, Fan et al. (14) revealed that lower health literacy was associated with higher nonadherence.

Conversely, some evidence shows negative or no association. In cardiovascular diseases, patients with inadequate health literacy skills were more likely to have low refill adherence, but this association becomes non-significant in multivariate model (15). Parmar et al. (8) assessed seven studies that investigated the association between health literacy and medication adherence in patients with type 2 diabetes among ethnic minorities. They reported that the association was weak and inconsistent. Similarly, Thurston et al. found no association in underserved patients with type 2 diabetes mellitus (16). Most of the evidence suggests a positive correlation or no correlation. These disparities in studies can be explained by differences in inclusion criteria, clinical settings, the use of heterogenous health literacy tools, differences in the measurement of adherence, disease and population variations, and confounding factors.

In this study we evaluated the association between health literacy and adherence to medication in patients with chronic medical conditions in the community pharmacy setting. Community pharmacy settings offer several advantages that other clinical settings do not provide. These include accessibility and convenience, high frequency of patient-pharmacy interactions, no-cost consultation, and less formal environment. Berenbrok et al. compared the number of patient visits to community pharmacies with those to the primary care physicians. Participants were classified into different subgroups based on demographics, region of residence, and clinical characteristics. The number of community pharmacy visits was significantly higher across all subgroups confirming the accessibility of healthcare professionals in community pharmacies. Hindi et al. assessed the views of patients on community pharmacy services in primary care. Patients stated that faster access and convenience was a very important value of using pharmacy services (17).

The association between health literacy and medication adherence within this specific setting and across different medical conditions has not been sufficiently investigated. This study aims to provide additional information that may guide the practices of pharmacists in community pharmacies.

## 2 Methods

This was a cross-sectional study conducted in the community pharmacy settings. The enrollment of patients stretched over 3 months from December 2025 to February 2026. Patients visiting the community pharmacies were approached for participation in the study. The inclusion criteria: any customer of the community pharmacy aged 18 years or older with at least one chronic medical condition for at least 3 months. Community pharmacies in the north, middle, and south of Jordan were used as a setting for the study to insure diverse geographical location that is not usually represented adequately in studies. In addition, several community pharmacies were used in the same geographical region to assure the inclusion of participants from different backgrounds and to have a representative sample. Convenient sampling was used to select pharmacies in different locations in the north, middle, and south of Jordan. Two pharmacies were selected in the south, 4 pharmacies in the north, and seven pharmacies in the middle of Jordan.

The questionnaire was distributed by the researchers and responsible pharmacists in the pharmacy by convenient sampling in a Google or paper form taking into consideration the environment of the pharmacy and rush hours. The participants were approached either in person or by phone calls. We could not calculate the response rate since participants were asked to circulate the questionnaire to family members within the same household who meet the inclusion criteria.

An ethical approval was obtained from the institutional review board (IRB) in the faculty of pharmacy - Applied Science Private University on the 18th of November 2025, number (025-PHA-36). The questionnaire was distributed in a Google form and provided to the participants through a QR code or filled on a paper form and transferred to the Google form by the researchers.

A cover letter at the beginning of the Google form contained the aim of the study with emphasis on the confidentiality and anonymity of the data. If patients agreed on participation in the study, they would start the questionnaire.

### 2.1 Study tool

The study tool consisted of five parts; sociodemographic information, clinical data, patient – community pharmacist interaction and counselling, adherence, and health literacy.

The Arabic validated version of Morisky Medication Adherence Scale (MMAS-8) was used to assess adherence, the was used in previous studies (18–20). It is a self-report 8 item questionnaire, with high reliability and validity, and utilized in many chronic conditions such as hypertension and diabetes mellitus (21–25). Medication adherence was categorized into three levels: low adherence (score <6), medium adherence (score 6 to <8), and high adherence (score = 8). Letter of permission for the use of Morisky Medication Adherence Scale (MMAS-8) was obtained with certificate number “MMAS8R-E18C79F8-EEFD2ED0-C0054AE2.”

The Arabic version of the Health Literacy Survey European Questionnaire’s short version (HLS-EU-Q16) was used for the assessment of comprehensive health literacy. It consists of 16 items with responses ranging from very difficult, difficult, easy, and very easy. Difficult/very difficult responses were assigned to the value of 0 and easy/very easy responses were assigned to the value of 1. A cumulative score of 0–8 is considered inadequate, 9–12 problematic, and 13–16 for sufficient health literacy (26).

### 2.2 Statistical analysis

The data were entered into and analyzed using the Statistical Package for Social Sciences (SPSS) version 22 (SPSS Inc., Chicago, IL USA). Descriptive statistics were used to summarize participant characteristics and presented categorical variables as frequencies and percentages, while continuous variables were presented as median and interquartile ranges (IQR).

To assess the relationship between adherence levels generated using the Morisky scale (MMAS-8) and the health literacy level generated using HLS-EU-Q16, Chi-square test of independence was used. To identify independent predictors influencing participants’ adherence level, logistic regression analysis was used. The predictor variables that had a *p*-value of <0.25 in the univariate analysis were selected for inclusion in the multivariate logistic regression model. For the purposes of this analysis, “medium” and “high” adherence levels were combined into a single response category to streamline the interpretation of these results. A *p*-value < 0.05 was considered statistically significant.

### 2.3 Sample size calculations


$$n=\frac{Z^{2}p(1-p)}{d^{2}}$$

Based on the above equation for observational studies, the sample size for the study was determined. *N* is the sample size, *Z* is the statistic corresponding to level of confidence *Z* for a 95% confidence interval is 1.96, *P* is expected prevalence assigned 50% which gives the largest sample size, d is precision (corresponding to effect size). *Z* = 1.96, *Z*^2^ = 3.8, *p* = 0.5, 1−*p* = 0.5, *d* = 0.05, *d*^2^ = 0.0025, *n* = 380 participants. However, additional number of participants were included to provide diversity in patient characteristics and views.

## 3 Results

This study included 502 participants, with a median age of 55.0 years (IQR = 18.0). Slightly more than half were males (*n* = 273, 54.4%), and the majority were married (*n* = 382, 76.1%). Additional details on the participants’ sociodemographic are presented in Table 1. Hypertension was the most common disease among the participants, followed by diabetes and dyslipidemia. Participants reported a median of 2.0 medical conditions (IQR = 2.0) and 3.0 medications per day (IQR = 2.0). Most participants rated their health as very good (*n* = 200, 39.8%) or good (*n* = 146, 29.1%), while 11.0% rated their health as fair, Table 2.

**Table 1:** Demographic characteristics of the study sample.

Parameter	Median (IQR)	*n* (%)
Age (years)	55.0 (18.0)	
Gender		
Male		273 (54.4)
Female		229 (45.6)
Marital status		
Married		382 (76.1)
None-married (single, divorced, widowed)		120 (23.9)
Educational level		
Primary		56 (11.2)
Secondary		90 (17.9)
Diploma		84 (16.7)
Bachelor		218 (43.4)
Higher education		54 (10.8)
Employment status		
Employed		228 (45.4)
Unemployed		159 (31.7)
Student		8 (1.6)
Retired		107 (21.3)
Family income (JD)		
<300		67 (13.3)
300–599		106 (21.1)
600–999		84 (16.7)
≥1,000		55 (11.0)
Prefer not to say		190 (37.8)
Geographic location		
North of Jordan		138 (27.5)
Middle of Jordan		283 (56.4)
South of Jordan		81 (16.1)
Residence		
Urban		417 (83.1)
Rural		85 (16.9)
Health insurance		
None		148 (29.5)
Public		241 (48.0)
Private		113 (22.5)

1 JD = 1.4 US dollar, IQR, interquartile range.

**Table 2:** Medical and health characteristics of the study sample.

Parameter	Median (IQR)	*n* (%)
Main chronic condition(s)^€^		
Hypertension		256 (51.0)
Cardiovascular disease (HF, IHD, ACS)		109 (21.7)
Dyslipidemia		241 (48.0)
Asthma		53 (10.6)
Diabetes		242 (48.2)
Others		140 (27.9)
Number of medical conditions	2.0 (2.0)	
Number of medications	3.0 (2.0)	
Number of tablets per day		
<5 per day		326 (64.9)
≥5 per day		176 (35.1)
Duration since first medical condition		
≤1 year		27 (5.4)
2–5 years		137 (27.3)
6–10 years		154 (30.7)
>10 years		184 (36.7)
Self-rated health		
Excellent		90 (17.9)
Very good		200 (39.8)
Good		146 (29.1)
Fair		55 (11.0)
Poor		11 (2.2)
Smoking status		
Current		182 (36.3)
Ex-smoker		116 (23.1)
Never		204 (40.6)

IQR, interquartile range; ^€^The sum of chronic medical conditions is more than 502 due to multiple comorbidities.

**Figure 1:**
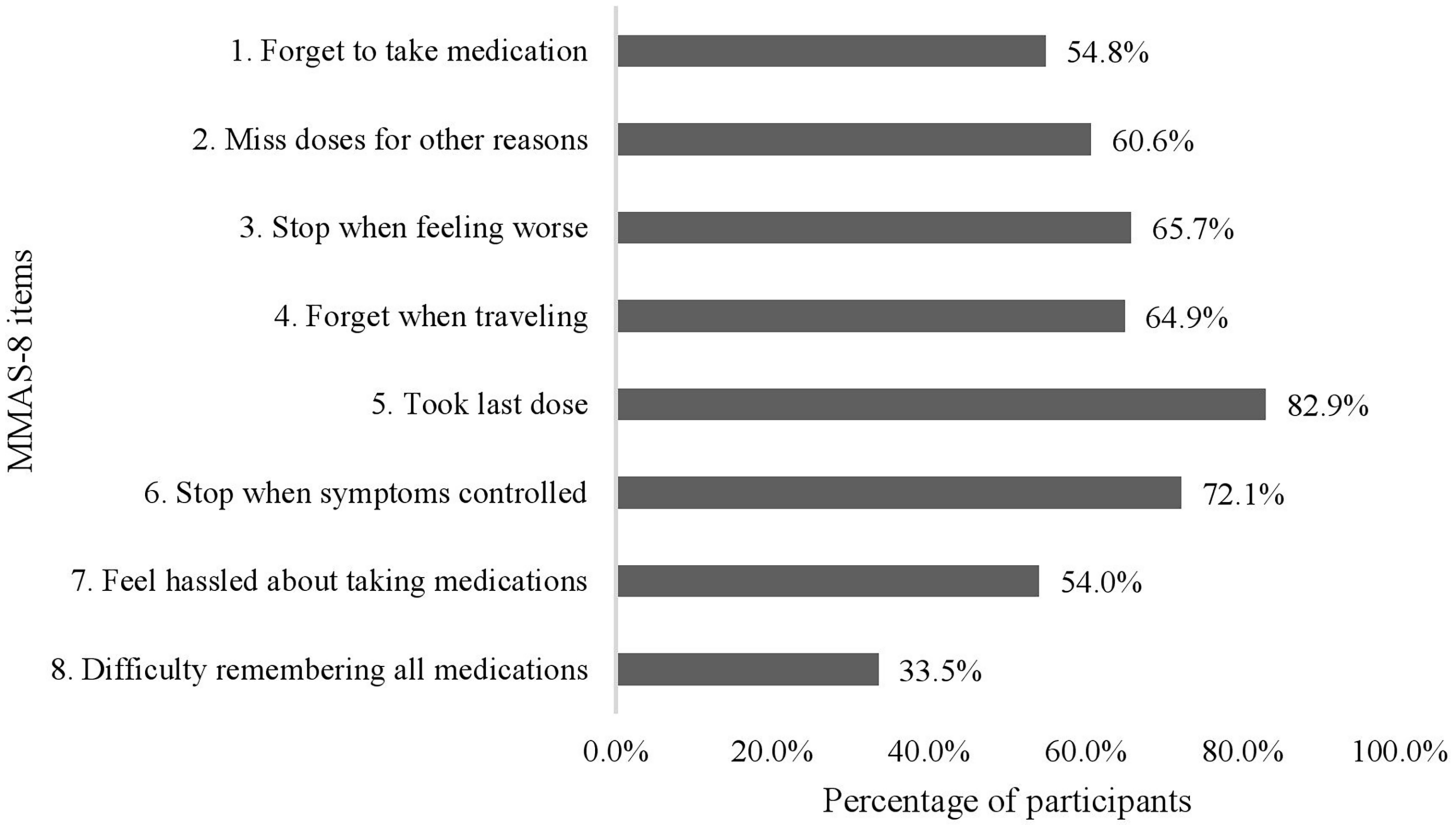
Proportion of participants reporting adherence behaviors for each MMAS-8 item (*n* = 502). Items 1–7 are yes/no questions where adherence is indicated by responses such as “No” to forgetting or stopping medication, except for item 5 (“Took last dose”) where “Yes” indicates adherence. Item 8 assesses frequency of difficulty remembering medications, with “Never” considered adherent responses.

Medication adherence was evaluated using the MMAS-8 scale. The highest adherence was observed for taking the last prescribed dose (*n* = 416, 82.9%), while the lowest adherence was reported for difficulty remembering all medications (*n* = 168, 33.5%) and forgetting to take medication (*n* = 275, 54.8%), Figure 1. Nearly half of the participants exhibited low adherence (*n* = 272, 54.2%), while 27.3% demonstrated medium adherence (*n* = 137). Only around one-fifth of the sample achieved high adherence (*n* = 93, 18.5%).

Patients’ interactions with community pharmacists as reported by the study participants showed that only one fifth of the participants received counselling at every refill. Counselling was most commonly conducted at the counter, and few participants reported private counselling and that verbal communication was the dominant preferred format for receiving information.

Most participants believed that the Teach-Back method is beneficial for improving understanding of medication use, Table 3.

**Table 3:** Patterns of patient-community pharmacist interaction and communication preferences.

Questions	*n* (%)
Frequency of pharmacist counselling	
Every refill	97 (19.3)
Sometimes	228 (45.4)
Rarely	113 (22.5)
Never	64 (12.7)
Privacy during counselling	
Counseling is conducted in a private area	47 (9.4)
Counseling is conducted at the counter	292 (58.2)
No counseling	64 (12.7)
Preferred format for information	
Verbal	398 (79.3)
Leaflet	57 (11.4)
Pictogram	11 (2.2)
SMS	22 (4.4)
Video	14 (2.8)
Do you believe that using a teach-back method by the pharmacist to check medication understanding is beneficial?	
Yes	452 (90.0)
No	23 (4.6)
I do not know	27 (5.4)

Health literacy of the study participants was assessed using the HLS-EU-Q16. Overall, participants demonstrated relatively high health literacy, with a median score of 14 (IQR = 5), indicating generally sufficient health literacy in the study population. The strongest performance was observed in interactions with healthcare professionals. The majority of participants reported ease in understanding medication instructions, understanding what the doctor says, and obtaining information on health treatments. In contrast, the most notable difficulty was observed in critical appraisal of health information, particularly judging the reliability of health information in the media. Additional challenges were identified in managing mental health information and deciding when a second medical opinion is needed, Table 4.

**Table 4:** Item-level health literacy (HLS-EU-Q16).

Statement	Difficult/very difficult *n* (%)	Easy/very easy *n* (%)
Find information on treatments of illnesses	51 (10.2)	451 (89.8)
Find where to get professional help when ill	74 (14.7)	428 (85.3)
Understand what your doctor says	48 (9.6)	454 (90.4)
Understand instructions on prescribed medicine	36 (7.2)	466 (92.8)
Judge need for second opinion	152 (30.3)	350 (69.7)
Use doctor’s information to make decisions	79 (15.7)	423 (84.3)
Follow instructions from doctor/pharmacist	51 (10.2)	451 (89.8)
Find information on managing mental health problems	161 (32.1)	341 (67.9)
Understand health warnings (smoking, activity, alcohol)	62 (12.4)	440 (87.6)
Understand need for health screenings	98 (19.5)	404 (80.5)
Judge reliability of health information in media	262 (52.2)	240 (47.8)
Decide how to protect yourself from illness using media information	109 (21.7)	393 (78.3)
Find activities for mental well-being	88 (17.5)	414 (82.5)
Understand advice from family or friends	73 (14.5)	429 (85.5)
Understand media information on how to get healthier	129 (25.7)	373 (74.3)
Judge everyday behaviors related to health	74 (14.7)	428 (85.3)

Most participants demonstrated sufficient health literacy (*n* = 330, 65.7%). A smaller proportion were classified as having problematic health literacy (*n* = 123, 24.5%), while inadequate health literacy was observed in 49 participants (9.8%).

Overall, adherence improved with increasing health literacy. Patients with inadequate health literacy had the highest proportion of low adherence (89.8%) and the lowest high adherence (2.0%). Those with problematic literacy showed slightly better but still predominantly low adherence (68.3%). In contrast, participants with sufficient health literacy demonstrated better adherence, with lower low adherence (43.6%) and higher medium (31.2%) and high adherence (25.2%), Table 5.

**Table 5:** Association between health literacy and medication adherence.

Health literacy level	Low adherence *n* (%)	Medium adherence *n* (%)	High adherence *n* (%)	Total	*p*-value^#^
Inadequate	44 (89.8%)	4 (8.2%)	1 (2.0%)	49	<0.001*
Problematic	84 (68.3%)	30 (24.4%)	9 (7.3%)	123	
Sufficient	144 (43.6%)	103 (31.2%)	83 (25.2%)	330	
Total	272 (54.2%)	137 (27.3%)	93 (18.5%)	502	

^#^Chi-square test of independence was used. *A statistically significant association was found between health literacy and adherence (*χ*^2^(4) = 54.92, *p* < 0.001).

Analysis of factors affecting participants’ adherence level showed that health literacy was a significant independent predictor, with participants having sufficient health literacy demonstrating higher odds of adherence compared to those with inadequate or problematic literacy, AOR = 2.730, 95% CI 1.764–4.224, *p* < 0.001. Participants living outside the middle region of Jordan had lower adherence compared to those in the middle region, AOR = 0.569, 95% CI 0.355–0.912, *p* = 0.019. Similarly, individuals reporting good, fair, or poor self-rated health had lower adherence than those with excellent or very good health, AOR = 0.590, 95% CI 0.383–0.909, *p* = 0.017, Table 6.

**Table 6:** Logistic regression analysis for factors affecting medication adherence.

Parameter	Dependent variable: adherence level [0: low, 1: medium/high]			
	COR (95% CI)	*p*-value^#^	AOR (95%CI)	*p*-value^$^
Age (years)	0.993 (0.980–1.006)	0.272	—	—
Gender				
Male	Reference			
Female	1.105 (0.777–1.572)	0.580	—	—
Marital status				
Married	Reference			
None-married (single, divorced, widowed)	0.802 (0.529–1.214)	0.291	—	—
Educational level				
Diploma or below	Reference			
Bachelor or above	2.014 (1.407–2.884)	<0.001^^^	1.333 (0.861–2.063)	0.197
Employment status				
Employed	Reference			
Others (unemployed, student, retired)	0.809 (0.596–1.152)	0.240^^^	0.985 (0.639–1.518)	0.946
Monthly income				
<600 JD	Reference			
≥600 JD	1.939 (1.327–2.834)	0.001^^^	1.202 (0.749–1.929)	0.445
Geographic location				
Middle of Jordan	Reference			
Others	0.493 (0.344–0.708)	<0.001^^^	0.569 (0.355–0.912)	0.019
Residence				
Urban	Reference			
Rural	(0.260–0.714)	0.001^^^	0.637 (0.356–1.143)	0.131
Health insurance				
No	Reference			
Yes	1.072 (729–1.577)	0.722	1.245 (0.810–1.913)	0.317
Number of medications	0.798 (0.720–0.884)	<0.001^^^	0.946 (0.776–1.153)	0.582
Number of chronic diseases	0.691 (0.588–0.814)	<0.001^^^	0.864 (0.659–1.132)	0.289
Number of tablets taken per day	0.531 (0.369–0.774)	0.001^^^	1.093 (0.584–2.048)	0.781
Duration since first medical condition				
<10 years	Reference			
>10 years	0.923 (0.641–1.330)	0.669	—	—
Self-rated health				
Excellent/very good	Reference			
Good/Fair/Poor	0.387 (0.267–0.559)	<0.001^^^	0.590 (0.383–0.909)	0.017*
Health literacy				
Inadequate/problematic	Reference			
Sufficient	3.758 (2.504–5.638)	<0.001^	2.730 (1.764–4.224)	<0.001*

COR, crude odds ratio, AOR, adjusted odds ratio, ^#^Using simple logistic regression, ^$^Using multiple logistic regression, ^^^Eligible for entry in multiple logistic regression, ^*^Significant at 0.05 significance level.

## 4 Discussion

Half of the participants exhibited low adherence, and several issues concerning adherence patterns emerged among the study population. For example, one third of the participants had problems remembering all their medication, half of them forget to take their medications, and most of them stop taking their medication when their symptoms are controlled. This is alarming, especially in a country with limited healthcare resources where failure of taking the medication after a long process of diagnosis, clinical visits, and dispensing medication is a waste of healthcare resources and drugs.

Abed et al. reported a higher rate of high adherence (54.3%) among Jordanian patients with multiple comorbidities but using another scale, the General Medication Adherence Scale (GMAS) (27). Patients with specific diseases demonstrated lower rates of adherence, for example, in patients with end-stage kidney disease, the mean of the Morisky Medication Adherence Scale (MMAS) score was 5.18 (SD = 2.024) (28).

Non-adherence due to stopping the medication after improvements in the medical condition is a behavior that can be managed by appropriate patient education delivered by the community pharmacists that focuses on the importance of continuing medication after improvement (29, 30).

Interactions with the community pharmacist revealed some interesting results. Privacy was absent in many of the communications with the pharmacist, counseling was inconsistent and mainly depended on verbal communications rather than videos or leaflets. Oral information, oral plus written information, visual aids, educational leaflets, organized counseling approach and follow up calls are all methods that can be used to provide counselling and improve adherence (31). Tailored interventions designed to meet the demand of various patients depending on their conditions and characteristics and the inclusion of different strategies for delivering the information demonstrated promising results on adherence (30). Privacy is a very important aspect of counseling, Alnesef et al. (32) assessed the patients` perceived level of privacy in polyclinic pharmacies in Kuwait. Although 63.8% of the patients expressed a high need for privacy, only 20% of participants reported a good to excellent score in patients’ privacy satisfaction. Alrasheed et al. (33) evaluated privacy in community pharmacies in Saudi Arabia. Almost half of the participants had concerns about privacy, the confidentiality of their health information, and refraining from discussing health concerns with the pharmacist due to privacy issues.

Participants in this study demonstrated relatively high health literacy, a possible explanation is that the education level of the participants was relatively high (70% had a diploma degree or higher). However, difficulties were noted in judging the reliability of health information in the media and managing mental health information. Health literacy was significantly associated with medication adherence and was a significant predictor of adherence, patients with inadequate health literacy had the highest proportion of low adherence. Hyvert et al. (13) reviewed 27 studies, they showed that the association between health literacy and adherence was not clear, and other factors may have a role. Lee et al. (34) demonstrated that health literacy was a strong predictor of medication adherence and they concluded that implementation of health literacy interventions may increase medication adherence and lead to better patient outcomes. Al-Qerem et al. (35) examined this correlation in Jordanian diabetic patients and came to the same conclusion. Higher health literacy scores significantly increased the likelihood of having high adherence level. Based on the previous evidence, improvement of health literacy presents as an opportunity for community pharmacist-led interventions that might be reflected on better adherence. Pao et al. (36) evaluated the effect of pharmacist-led multidisciplinary care versus standard care on health literacy. The intervention group had a 21.1% increase in health literacy that was accompanied by a similar increase in adherence. Cork et al. (37) investigated the opinions of community pharmacists regarding the extent of practicing health literacy interventions. The assessed interventions included Teach-Back, Chunk and Check, simple language and visual aids. The most popular method was Teach-Back because it is suitable for all ages and does not consume time. Visual aids were used with Teach Back and it was challenging to use simple words rather than medical terms. Community pharmacists should be acquainted with their role in improving health literacy in practice. Equally important, patients should be aware of the pharmacist’s role and their effect on health literacy which might be overlooked or underestimated due to the superficial and quick encounters with the community pharmacist. Adapting a patient-centered care approach, finding solutions for time management, and behavioral change in patients that drives them to take more responsibility over their health are opportunities for improving health literacy. Additionally, pharmacists should be proactive in implementing tools and methods that are already known to improve health literacy.

Factors that predicted adherence included health literacy, geographical location, and the patient’s own perception of their health. Geographic location may reflect socioeconomic and cultural differences. Even in countries with better healthcare services, geographic location was among the factors that determined the likelihood of being adherent to medication (38). Similar to our results, Liu et al. reported that self-rated health status and living area were associated with full and partial adherence (39). Han et al. (40) investigated environmental and individual influences on adherence in hypertensive patients with chronic kidney disease. Patients who reside in medically underserved areas had poor medication adherence. In terms of perceived health status, Xie et al. (41) demonstrated that patients with better perceived health status were more likely to adhere to medication. Abdelhamid et al. (42) investigated medication adherence and illness perception in diabetic patients. Low/medium illness perception, compared to high perception, predicted low adherence.

These findings justify tailored, regionally relevant interventions to improve medication adherence taking into consideration the needs of different communities in the same country. Chauke at al. (43) systematic review revealed other factors in low- and middle-income countries such as lack of knowledge, negative attitudes and beliefs influenced unfavorably adherence. Gender impact on adherence is conflicting, Bąk-Sosnowska et al. (44) showed that females had higher adherence than males, however, Lewey et al. (45) reported that women had 10% lower odds of adherence than men. Barakat et al. (46) findings underscored other factors that affected adherence such as age, education, income, smoking, and insurance in Jordanian patients with dyslipidemia in hospital setting.

Evidence shows that community pharmacists can play an important role in improving adherence. Oñatibia-Astibia et al. (47) review of four studies that included 2,266 patients revealed that interventions provided by community pharmacists can improve adherence to lipid lowering medications. Milosavljevic assessed twenty-two studies that investigated the impact of community pharmacist-led interventions on patients’ adherence. Their findings showed that community pharmacist-led interventions improved patients’ adherence which was reflected on better clinical outcomes in hypertension, dyslipidemia, and chronic obstructive pulmonary disease but not on diabetes or depression (48). Studies evaluating adherence should be interpreted carefully taking into consideration the methods used in assessing the magnitude of adherence since there are no guidelines or recommendations on choosing the appropriate tools which can be reflected on the findings (49).

This study evaluated the link between health literacy and adherence in a community pharmacy setting in three main geographical regions in the country which allowed the detection of geographical location as a significant predictor of adherence and highlighted the need for region-specific pharmacy interventions rather than one-size-fits-all approaches.

The study has several limitations; we assessed the correlation between demographic characteristics and health literacy with adherence. However, other patient related factors affect adherence such as the psychological factors. Patients with lower stress levels, a belief that one’s behavior affects his/her health condition, and fully being aware of one’s actions have better adherence (44). Additionally, the cross-sectional design does not establish causality between health literacy and adherence. Pharmacy-based convenience sampling includes patients attending pharmacies which may over-represent adherent individuals compared to the broader chronic disease population. Social desirability bias is another limitation since MMAS-8 is a self-report scale, and patients are known to over-report adherence. Moreover, no objective adherence validation was done, there was no pill count, refill records, or biomarkers to confirm self-reported adherence. Finally, non-randomized pharmacy selection limits the generalizability of the findings.

In conclusion, our findings revealed that more than half of the participants demonstrated low medication adherence which demonstrates the persistent challenge of medication non-adherence in the management of chronic diseases. Most participants demonstrated sufficient health literacy, but gaps in evaluating the reliability of media-based health content exist. Findings support the existing evidence that links health literacy to adherence behavior and justifies addressing literacy-related barriers in pharmacy practice. Geographical location and self-rated health status were identified as independent predictors of adherence, which suggests that adherence is a multifaceted behavior. Most participants favored the Teach-Back method for improving medication understanding indicating that structured communication is acceptable. Future longitudinal studies are needed to establish causal relationship and region-specific investigations to identify adherence patterns across the regions of Jordan. Additionally, it should explore the point of view of community pharmacists’ perception of their role, knowledge, and implementation of interventions that improve health literacy.

## Data Availability

The raw data supporting the conclusions of this article will be made available by the authors, without undue reservation.
